# Aggressiveness Potential of Spontaneous Canine Mucosal Melanoma Can Dictate Distinct Cancer Stem Cell Compartment Behaviors in Regard to Their Initial Size and Expansion Abilities

**DOI:** 10.1089/scd.2019.0223

**Published:** 2020-07-09

**Authors:** Yasmine Touil, Zacharie Segaoula, Xavier Thuru, Sylvie Galiègue-Zouitina, Dominique Tierny, Bruno Quesnel

**Affiliations:** ^1^ University of Lille, CNRS, Inserm, CHU Lille, Institut Pasteur de Lille, UMR 9020, UMR-S 1277 – Canther – Cancer Heterogeneity, Plasticity and Resistance to Therapies, Lille, France.; ^2^ OCR (Oncovet Clinical Research), SIRIC ONCOLille, Parc Eurasante, Loos, France.; ^3^ Oncovet Cancer Centre, Villeneuve d'Ascq, France.

**Keywords:** melanoma, stem cells, comparative oncology

## Abstract

Mucosal melanoma represents one of the most highly metastatic and aggressive subtypes of melanoma. The biology of mucosal melanoma is poorly documented, and the lack of experimental models makes it difficult to design and test new therapies. Dogs are frequently affected by melanomas of the oral cavity, making spontaneous canine melanoma a potentially predictable model for their human counterpart. We recently established and characterized two new canine mucosal melanoma cell lines named OCR_OCMM1 and OCR_OCMM2. Here, we identified quiescent cancer stem cell (CSC) subpopulations in both canine cell lines that displayed similarities to human quiescent CSCs: canine melanoma CSCs had the ability to self-renew, produced nonstem cell (SC) progeny, and formed melanospheres that recapitulated the phenotypic profile of the parental tumor. These CSCs also formed melanoma in immunodeficient mice, and the inhibition of PI3K/AKT signaling expanded the CSC pool. A subset of non-CSCs transitioned to become CSCs. OCR_OCMM1 and OCR_OCMM2 displayed different CSC compartment behaviors in regard to their initial size and expansion abilities. Collectively, this study showed that the OCR_OCMM1 and OCR_OCMM2 canine melanoma cell lines are powerful cellular tools to study melanoma SCs, not only for mucosal but also for the more common human cutaneous melanoma.

## Introduction

Increasing evidence suggests that melanoma cancer stem cells (CSCs) play an essential role in the resistance as well as in the relapse of this disease [1–3]. CSCs are able to enter quiescence, preserving their self-renewal capacities and, in this way, escaping from the current anticancer treatments, which usually target cycling cells. In our previous studies, we highlighted the regulation of human melanoma CSCs through the PI3K/AKT pathway [4]. In response to stress or to the modification of their niche or microenvironment, these cells showed extraordinary plasticity by quickly switching their phenotypic balance between quiescent and cycling states, thus making these cells difficult to specifically target. In addition, we also observed in a mouse model of melanoma dormancy that long-term persistent cancer cells showed characteristics of CSC [5].

Despite these important findings, the relevance of the currently available preclinical melanoma mouse models is questioned, notably because of the size and life span differences between rodents and humans. Accumulating data have highlighted the translational potential of veterinary spontaneous cancer models for elucidating the behaviors of many human cancers [6], including melanoma [7]. Several similar biological and clinical features have been reported between canine melanoma and the corresponding human cancer [8]. In humans, mucosal melanoma of the oral cavity is rare but is highly aggressive and metastatic [9], with frequent relapse and poor outcomes [10]. Recently, we isolated and characterized two canine oral melanoma cell lines (OCR_OCMM1 and OCR_OCMM2) from spontaneous mucosal melanoma tumors from canine patients with distinct clinical profiles and metastasis patterns [11]. The OCR_OCMM1 and OCR_OCMM2 melanoma cell lines had similar morphological, histological, and DNA copy number alteration (CNA) features to the original tumor and showed similar resistance/sensitivity to current antimelanoma drugs that are used for humans, suggesting the relevance of these cell lines as powerful research tools for human and canine medicine [11].

In the present study, we characterized subpopulations of cells with stem-like in vitro and in vivo properties in the OCR_OCMM1 and OCR_OCMM2 cell lines. The canine CSCs displayed similar functional characteristics to human melanoma CSCs and were also regulated by the PI3K/AKT pathway. These results reinforce the interest in veterinary spontaneous melanoma models as translatable preclinical tools that could improve antimelanoma therapies for humans in the future.

## Materials and Methods

### Reagents

Rhodamine 123 (Rh123), diamidino-2-phenylindole (DAPI), and DiI were purchased from Molecular Probes (Leiden, The Netherlands). Propidium iodide (PI), trypsin/EDTA, paraformaldehyde (PAF) solution, and poly-2-hydroxyethylmetacrylate were purchased from Sigma–Aldrich (Saint-Quentin-Fallavier, France). LY294002 was purchased from Calbiochem (France), epidermal growth factor was purchased from Stem Cells Biotech (Vancouver BC, Canada), and recombinant human basic fibroblast growth factor was purchased from PromoKine-PromoCell GmbH (Heidelberg, Germany).

### Canine melanoma cell lines, spheres, and culture

Two canine melanoma cell lines, OCR_OCMM1 and OCR_OCMM2, were isolated from melanoma tumor tissues obtained from a 14-year-old Yorkshire Terrier and an 11-year-old German Shepherd, respectively, that presented melanocytic lesions of the oral cavity, as previously described [11]; only the latter dog had metastasis to the lungs. The storage and use of canine biological samples were declared and were performed according to ethical rules approved by the Department of Health, France. The A375 human melanoma cell line was used as a control and was purchased from the American Type Culture Collection (ATCC^®^ CRL-1619). The OCR_OCMM1, and OCR_OCMM2 and A375 cell lines were cultured at 37°C in a humidified atmosphere and a 5% CO_2_ environment in RPMI supplemented with 10% fetal calf serum (FCS). The medium was changed every 3 days.

Spheres were generated in 24-well plates, as previously described [4]. When mentioned, cells were stained with 1.0 μM DiI fluorescent dye for 10 min at 37°C in PBS and then were rinsed and plated as described above. Sphere cultures were fed every 5 days by adding a 1:1 volume of fresh medium, and sphere-forming unit (SFU) values were estimated after 7–14 days. Spheres were counted under a microscope by two independent experimenters. For the self-renewal assay or in vivo experiments, primary spheres were recovered, dissociated by a short trypsinization, and were replated at a clonal density of 1,000 cells/mL.

### Flow cytometry

Adherent cells or spheres containing slow-cycling DiI^high^ cells were trypsinized or dissociated, respectively, and single-cell suspensions (2 × 10^5^ cells/500 μL) of OCR_OCMM1 or OCR_OCMM2 cells were incubated for 30 min on ice in 100 μL of RPMI with a primary anti-ABCB5 antibody (Ab; Rockland, Tebu-Bio, France), which was used at a 1:215 dilution. After incubation, the cells were rinsed with RPMI, centrifuged, and resuspended in 100 μL of RPMI with a secondary Ab, APC goat anti-rabbit IgG (H + L) (Molecular Probes^®^, Life Technology™), which was used at a 1:2,000 dilution for 30 min on ice in the dark. After incubation, the cells were rinsed with RPMI, centrifuged, resuspended in 500 μL of RPMI, and placed on ice before being analyzed by flow cytometry. PI-positive dead cells were gated out and excluded from the analysis.

Data acquisition was performed on an EPICS-CYAN flow cytometer (Beckman Coulter France S.A.S.), and data were analyzed using Summit 4.3 software. The DiI and APC fluorescence intensities were recorded on the FL2 and FL8 channels, respectively. Quadrants were determined based on the negative control staining with a corresponding isotype Ab. Fluorescence-activated cell sorting (FACS): cell sorting was performed on a FACS ARIA III sorter (Beckman Coulter France S.A.S.). Spheres were dissociated, and the single-cell suspension was adjusted to a concentration of 10^6^ cells/mL in RPMI. After excluding cell debris, the collection gates were set according to the negative (DiI-negative) control cells. Cells with positive fluorescence corresponded to the DiI-positive cell subset. The collected cells were centrifuged, rinsed, and replated for sphere, cell cycle analysis, and in vivo experiments.

### Rh123 exclusion assay

Cells were trypsinized, counted, and adjusted to a concentration of 10^6^ cells/mL in RPMI with 10% FCS. An Rh123 exclusion assay was performed, as previously described [4]. When indicated, cells were either pretreated with 10 μM LY294002 for 30 min at 37°C before Rh123 loading or pretreated and treated for 20 min of the Rh123 loading (50 min of exposure). After removing Rh123, a 60-min exclusion step was performed in the presence or absence of LY294002. After FACS, collected cells were plated for sphere generation and cell cycle analysis.

### Evaluation of cell growth

Population doubling times (PDTs) were evaluated for each cell line in adherent and spheroid conditions. Cells were plated at a density of 1 × 10^5^ cells in 60-mm petri dishes with 4 mL of complete growth medium and were then incubated at 37°C in a controlled-humidity atmosphere with 5% CO_2_ for 96 h. Cells were trypsinized (0.25% EDTA) and counted every day in duplicate, and the PDT was calculated using the following formula: PDT = 1/[3.32(logNH − logNI)/(t2 − t1)] (where t1 = time in hours when cells were seeded; t2 = time in hours when cells were harvested; NI = cell count when cells were seeded; and NH = cell count when cells were harvested), as previously described [11].

### Immunohistochemistry

Spheres or pieces of tumors were embedded in Tissue-Tek OCT (Sakura FineTechnical, Tokyo, Japan) and were stored at −80°C until cryosectioning. Sections (10 μm) were placed on glass slides and were air-dried and fixed in PAF solution, and immunohistochemistry was performed according to standard procedures. A monoclonal anti-M-CAM and Melan-A Abs and rabbit polyclonal anti-Nestin and anti-OCT4 Abs were purchased from Novus and were used at a dilution of 1/100. Secondary Alexa Fluor 488 or 594 goat anti-mouse or anti-rabbit Abs (Life Technologies, Cergy-Pontoise, France) were used at a dilution of 1/1,000. Negative controls were generated by replacing primary Abs with irrelevant Abs of the same isotype. DAPI were used for nuclear counterstaining.

All slides were mounted under a coverslip and were photographed using a Leica DMRB microscope (Leica Microsystems, Wetzlar, Germany) with a PL Plan-Leica Fluotar 20 × /1.00 objective. Photographs were taken with a DFC345FX digital camera and were processed with the Leica Application Suite (LAS v 3.7 software; Leica Microsystems), and images were further processed using the ImageJ^®^ software.

### Cell cycle analysis

The cell suspension was rinsed twice with PBS and fixed with 70% ethanol at −20°C. After rinsing with cold PBS, cells were incubated with 50 μg/mL PI and 5 μg/mL RNase in PBS for 30 min at room temperature. To determine the percentage of cells in the G0 phase of the cell cycle, cells were preincubated with an anti-human APC-conjugated Ki67 Ab (Abcam, Paris, France) or with a matching isotype on ice for 30 min before adding PI and RNase.

### In vivo tumorigenesis studies

Severe combined immunodeficient Hairless Outbred (SHO^®^) nude mice were purchased from Charles River (Wilmington, MA) and were kept under specific-pathogen-free conditions in individual IVS-type cages in the core animal facility of PLETHA 193 (Institute Pasteur de Lille, France). All protocols were approved by the institutional animal care and ethics committee and were in accordance with the European parliament directive (2010/63/EU). Five thousand sorted DiI^high^ or DiI^low^ cells from spheres were mixed with Matrigel solution and were subcutaneously (s.c.) injected at the interscapular site of 8-week-old mice (*n* = 8). Tumor growth was monitored every 3–4 days. Tumor volume was evaluated using a caliper, and the volume was calculated as 0.5 × length × width^2^.

### Statistical analyses

The results are expressed as the mean ± standard error of the mean, and statistical analyses were performed using GraphPad 320 Prism^®^ and StatView^®^ 5.0 software (SAS, Cary, NC). Data were analyzed using analysis of variance, followed by Fisher's protected least significant difference post hoc test, with a significance level of *P* < 0.05 (*P < 0.05; **P < 0.01; ***P < 0.005; and ****P < 0.001).

## Results

### Identification of a slow-cycling cell subpopulation with stem cell markers in canine melanoma cell lines

**FIG. 1.**
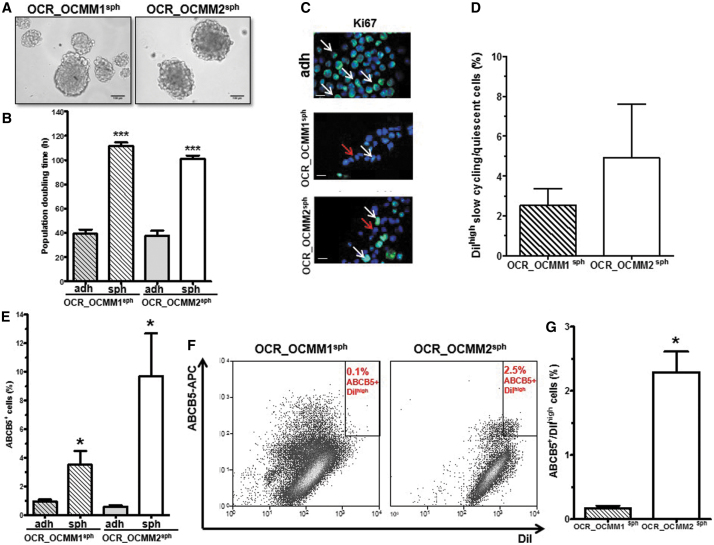
Identification of a slow-cycling cell subpopulation with SC markers in canine melanoma cell lines. **(A)** OCR_OCMM1 (*left*) and OCR_OCMM2 (*right*) cells were plated at a clonal density of 1,000 cells/mL and were grown in sphere-forming conditions. After 7 days of culture, large tumor-like spheres formed, and images were taken with an inverted microscope. Scale bar, 100 μM. **(B)** Spheres divided slower than adherent cells. ***P < 0.005. The PDT was calculated as described in the Materials and Methods section for spheres (sph) and adherent cells (adh). **(C)** Immunostaining of adherent and spheroid OCR_OCMM1 and OCR_OCMM2 cells for Ki67 expression (*green*). Nuclei were stained with DAPI (*blue*). Scale bar, 50 μM. The *arrows* indicate Ki67-positive cells, and the *red arrows* indicate Ki67-negative (quiescent) cells. **(D)** Histograms showing the percentage of DiI^high^-labeled slow-cycling/quiescent cells in OCR_OCMM1 and OCR_OCMM2 spheroids. **(E)** Spheroids were enriched for ABCB5^pos^ cells compared with adherent conditions. *P ≤ 0.05. **(F)** Representative flow cytometry graphs of ABCB5^pos^DiI^high^ cells (CSCs) in OCR_OCMM1 (*left*) and OCR_OCMM2 (*right*) spheroids. **(G)** Their quantification is represented with histograms. *P ≤ 0.05. CSC, cancer stem cell; DAPI, diamidino-2-phenylindole; PDT, population doubling times; SC, stem cell.

Sphere formation ability has been shown to define the stem cell (SC) compartment among from the total cell population in several tumor models, including in melanoma [12,13]. In addition, CSCs are mostly quiescent and rarely divide, displaying a slow cell-cycling profile. OCR_OCMM1 and OCR_OCMM2 canine melanoma cells were able to generate melanospheres, as shown in Fig. 1A. Seven days after plating, the melanospheres were quantified (SFUs = 0.4% ± 0.02% and 1.7% ± 0.06%, respectively). As shown in Fig. 1B, in both cell lines, cells isolated from spheres divided dramatically slower (2.5 times) than their adherent counterparts, indicating that there was an enrichment of slow-cycling cells in spheroid culture conditions.

A comparative immunohistofluorescence analysis between melanospheres and adherent cells revealed a higher proportion of KI67-negative cells in spheres than in adherent cells (Fig. 1C). Supplementary Figure S1 shows that both spheres and adherent cells exhibited similar positivity levels for the invasion and metastasis marker M-CAM, the embryonic marker of pluripotency and self-renewal OCT4, as well as for the SC marker NESTIN.

The slow-cycling of CSCs allowed the characterization of CSCs as label-retaining cells (LRCs) [14,15]. As shown in Fig. 1D, the presence of DiI^high^ cells or LRCs in melanospheres confirmed the presence of slow-cycling cells in melanospheres from both cell lines.

In addition, the well-characterized melanoma SC marker ABCB5 [16–18] was assessed by flow cytometry in spheres and adherent cells. ABCB5-positive cells were significantly enriched in spheres compared with adherent cells in OCR_OCMM1 and OCR_OCMM2 canine melanoma cells (3.5- and 9.5-fold enrichment, respectively, Fig. 1E). The presence of ABCB5^pos^ DiI^high^ cells, reflecting a quiescent/slow-cycling melanoma SC compartment, was also confirmed by flow cytometry in spheroid culture conditions (Fig. 1F). The OCR_OCMM2 canine melanoma cell line had more ABCB5^pos^DiI^high^ cells than the OCR_OCMM1 cell line (Fig. 1G). Collectively, our data identified a subpopulation of quiescent/slow-cycling SCs in our two canine melanoma cell lines.

### Identification of Rh123^low^ quiescent cells with stem-like properties

**FIG. 2.**
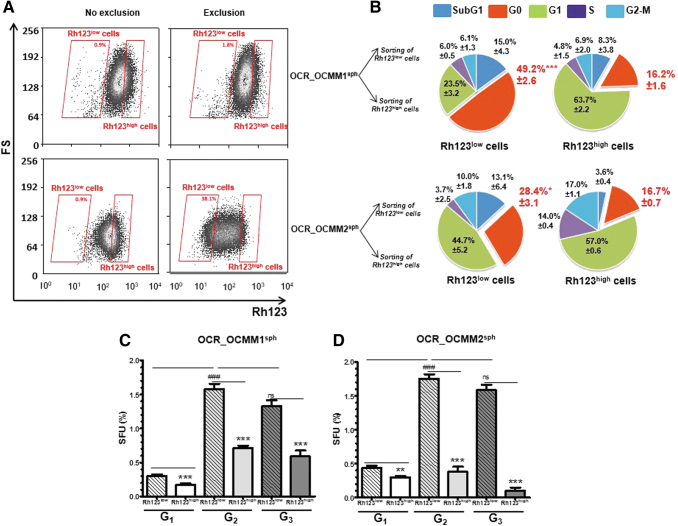
Identification of Rh123^low^ quiescent cells with stem-like properties. **(A)** After dissociation, OCR_OCMM1 and OCR_OCMM2 spheroid cells were incubated with 0.1 μg/mL Rh123 for 20 min (*left panel*). After 60 min of Rh123 exclusion, a subpopulation of low Rh123-retaining (Rh123^low^) cells appeared (*right panel*). **(B)** Rh123^low^ and Rh123^high^ cells were sorted by FACS, and the cell cycle was assessed by flow cytometry after KI67/PI staining. A fraction of cells in the G0 phase of the cell cycle is shown in *red*. *P ≤ 0.05 and ***P ≤ 0.005. OCR_OCMM1 **(C)** and OCR_OCMM2 **(D)** Rh123^low^ and Rh123^high^ fractions were sorted by FACS, replated at a clonal density of 1,000 cells/mL, and grown under sphere-forming conditions. After 7 days of culture, large tumor-like spheres formed, mainly from the Rh123^low^ fraction (quantitative graph below). The self-renewal capacity of melanospheres was assessed by dissociating and replating the cells at a clonal density for three successive generations (G_1_–G_3_). **P ≤ 0.01; ***P ≤ 0.005; and ^###^*P* ≤ 0.005 (* and # for the comparison of Rh123^low^ vs. Rh123^high^ and G_n_ Rh123^low^ vs. G_n+1_ Rh123^low^, respectively). FACS, fluorescence-activated cell sorting; PI, propidium iodide; Rh123, rhodamine 123; SFU, sphere-forming unit.

We previously used the functional Rh123-efflux assay to identify human melanoma [4] quiescent SCs in a subpopulation of cells with reduced Rh123 retention ability (Rh123^low^ cells). Rh123 is a substrate for ABCB1 and ABCB5 transporters [18,19], and low Rh123 fluorescence occurs as a result of high drug efflux activity. To determine whether canine melanomas also contain an Rh123^low^ subset, cells from OCR_OCMM1 and OCR_OCMM2 spheroids were assessed for their Rh123 efflux ability. After 1 h of incubation in dye-free medium, a subpopulation of Rh123^low^ cells appeared (Fig. 2A, right panel), suggesting that the Rh123 efflux in these cells was ABCB1/ABCB5-dependent. The OCR_OCMM2 melanospheres had a larger Rh123^low^ cell subpopulation than the OCR_OCMM1 spheres (30.63% ± 3.7% and 3.6% ± 0.5%, respectively). Together with the results in Fig. 1E and G, these results suggest that there were more SC-like cells among the OCR_OCMM2 cells than among the OCR_OCMM1 cells.

As the Rh123 exclusion functional assay allowed us to determine the enrichment of melanospheres in quiescent SCs, we performed cell cycle analysis with PI DNA staining and an anti-Ki67 Ab that recognizes cycling but not G0 quiescent cells. As expected, in spheres from both canine melanoma cell lines, more G0 quiescent cells (49.2% and 28.4%) were detected in the Rh123^low^ subset than in the Rh123^high^ compartment (16.2% and 16.7%, respectively) (Fig. 2B and Supplementary Fig. S2). Interestingly, there were more cells in the G0 state in OCR_OCMM1 Rh123^low^ cells than in OCR_OCMM2 Rh123^low^ cells (49.2% and 28.4%, respectively). As the OCR_OCMM2 Rh123^low^ cell population was larger than the OCR_OCMM1 Rh123^low^ cell population (30.63% ± 3.7% and 3.6% ± 0.5%, respectively), it appears that the stem-like compartment in OCR_OCMM2 spheroids was predominantly composed of G1-activated stem-like cells, whereas OCR_OCMM1 Rh123^low^ cells were predominantly composed of G0-quiescent cells. The significance of this observation is not yet known, but it may correspond to the differences in the metastatic potential of the melanoma in these two dogs [11].

A functional analysis of sorted OCR_OCMM1 and OCR_OCMM2 Rh123^low^ spheroid cells revealed that this fraction formed two times more first-generation (G_1_) spheres after sorting than their Rh123^high^ counterparts (Fig. 2C, D) [12]. Melanosphere cells formed new spheres for a minimum of three consecutive generations (Fig. 2C, D), demonstrating their self-renewal and long-term repopulating abilities. Of note, for both the OCR_OCMM1 and OCR_OCMM2 cell lines, the passage of G_1_ Rh123^low^ spheroid cells to G_2_ spheres was accompanied by a 5- and 4.25-fold increase, respectively, in the SFU values (Fig. 2C, D), although the same number of live cells was plated for the sphere-forming assay at each generation. These results can be explained by the expansion of the SC compartment in sphere cultures. However, the sphere-forming capacity of the Rh123^low^ cells in the third generation was not significantly different from that in the second generation, indicating that the expansion abilities of stem-like cells were limited to G_1_ spheres, and the cells mostly undergo self-renewal in subsequent generations.

In contrast to Rh123^low^ cells, the sphere-forming ability of non-SC Rh123^high^ spheroid cells significantly decreased with each spheroid generation. This phenomenon was observed in the two canine cell lines but was more pronounced in the OCR_OCMM2 cell line. Indeed, in the third generation, SFU values reached 0.1% for Rh123^high^ cells from OCR_OCMM2 spheroids and 0.6% in the other melanoma canine cell line, indicating an exhaustion of the propagative and sphere-forming capacities of the Rh123^high^ cells; these data confirmed the close relationship between Rh123^low^ cells and the characteristic sphere-forming and self-renewal abilities of SCs.

Accordingly, cell cycle analysis of sorted OCR_OCMM2 Rh123^high^ cells showed more cells in S phase and G2/M phase than in the OCR_OCMM1 cells (14% and 17% vs. 5% and 7%), reflecting a higher proportion of dividing cells in the non-SC Rh123^high^ cell compartment of the OCR_OCMM2 cell line. Collectively, our results demonstrate the presence of an Rh123^low^ cell subpopulation with stem-like characteristics, such as quiescence and self-renewal capacity, in both canine melanoma cell lines and show that the Rh123 functional assay is a valuable tool for differentiating the cancer stem-like and non-SC compartments.

### Inhibition of the PI3K/AKT pathway increased the stem-like cell pool

**FIG. 3.**
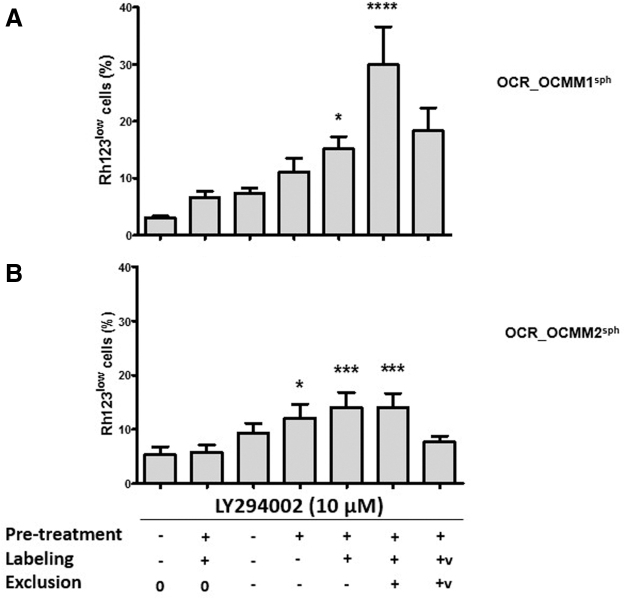
Time- and ABC transporter-dependent effects of LY294002 on the size of the Rh123^low^ pool. OCR_OCMM1 **(A)** and OCR_OCMM2 **(B)** sphere cells were treated with 10 μM of LY294002 (+) or growth medium (−) for different durations during the Rh123 dye exclusion assay (pretreatment-30 min; labeling-20 min; and exclusion-60 min) (*n* = 8). A final concentration of 50 μM verapamil (V) was added as a control where indicated; (0) indicates no exclusion. *P ≤ 0.05; ***P ≤ 0.005; and ****P ≤ 0.001.

We previously showed that the PI3K/AKT signaling pathway controls the oscillation between the active and quiescent states of Rh123^low^ cells with SC-like characteristics; therefore, this signaling pathway controls the size of the Rh123^low^ melanoma SC compartment in human melanoma [4]. Since we also confirmed these observations in the human A375 melanoma cell line (Supplementary Fig. S3), we questioned whether the PI3K/AKT signaling pathway also plays a crucial role in the regulation of Rh123^low^ stem-like cells in our two canine melanoma cell lines. OCR_OCMM1 and OCR_OCMM2 cells were treated with LY294002, an inhibitor of the PI3K/AKT signaling pathway, 50 min before Rh123 efflux stage. As shown in Fig. 3, a short LY294002 treatment significantly expanded the pool of Rh123^low^ cells.

The size of the Rh123^low^ pool was directly proportional to the duration of LY294002 exposure. A short exposure to LY294002 specifically upregulated the functionality of multidrug resistance transporters in both canine melanoma cell lines, and this change correlated with the Rh123^low^ phenotype. The observed expansion in the Rh123^low^ subset was very rapid and occurred during the 60-min Rh123 exclusion step. During this time, neither cell death nor cell division could significantly change the size of the total cell population. Therefore, most of the new Rh123^low^ cells likely came from Rh123^high^ cells shifting to an Rh123^low^ phenotype by activating their dye exclusion machinery. Thus, the inactivation of the PI3K/AKT pathway stimulated ABCB1/ABCB5 transporter functionality, contributing to a phenotype switch and to the expansion of the Rh123^low^ melanoma SC compartment, as was previously shown for human melanoma (Touil et al. [4] and Supplementary Fig. S3). The fold increase in the Rh123^low^ subset during the 60-min exclusion step was significantly higher in the OCR_OCMM1 cell line than in the OCR_OCMM2 cell line (4.3-fold vs. 1.4-fold increase, respectively). These results demonstrated that among Rh123^high^ cells, a subpopulation of melanoma cells is capable of reverting to SCs with an Rh123^low^ phenotype. The OCR_OCMM1 cell line contained a larger pool of these cells than OCR_OCMM2, as the OCR_OCMM1 cell line had a larger number of Rh123^high^ cells in the G1 phase of the cell cycle, suggesting that there are cells capable of phenotypic switching among these particular cells.

### Slow-cycling cells from melanospheres induce tumor formation in immunodeficient mice

**FIG. 4.**
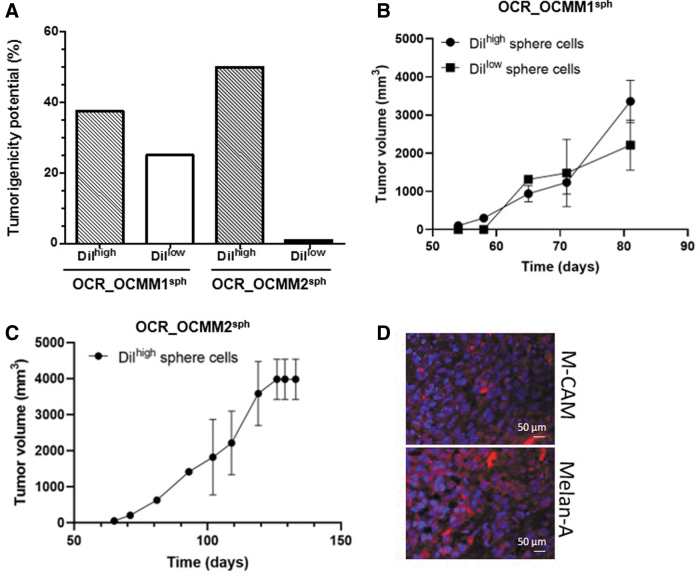
Slow-cycling cells, DiI^high^ LRCs, from melanospheres induce tumor formation in immunodeficient mice. DiI^high^ LRC slow-cycling cells from OCR_OCMM1 and OCR_OCMM2 spheroids had a higher tumorigenic potential than their DiI^low^ highly proliferative counterparts. Nude mice were s.c. injected with 5,000 DiI^high^ or DiI^low^ cells. **(A)** The results are expressed as the percentage of mice bearing tumors relative to the total number of injected mice. The histograms represent the mean ± standard error of the mean of two independent experiments. Graph showing the tumor sizes at each time point for OCR_OCMM1 **(B)** and OCR_OCMM2 **(C)** conditions. Tumor size was evaluated as described in the Materials and Methods section. **(D)** M-CAM and Melan-A expression was evaluated by immunostaining of tumor sections generated from DiI^high^ spheres cells. Nuclei were stained with DAPI. Scale bar, 50 μM. LRCs, label-retaining cells; s.c., subcutaneously.

As the OCR_OCMM1 and OCR_OCMM2 canine melanoma cell lines had quiescent/slow-cycling SCs with an ABCB5^pos^DiI^high^ status, we examined whether this cell subpopulation was capable of forming tumors in vivo with a higher efficiency than their non-SC counterparts. As shown in Fig. 4A, 5,000 sorted DiI^high^ and DiI^low^ cells from OCR_OCMM1 and OCR_OCMM2 spheroids were s.c. injected into immunodeficient mice and displayed substantial tumor formation potential (37.5% and 50% vs. 25% and 0%, respectively, for both canine melanoma cell lines).

Notably, the sorted DiI^high^ OCR_OCMM2 spheroid cells were more tumorigenic than OCR_OCMM1 spheroid cells, as they developed spheres more efficiently (Fig. 2C, D). These results correlated well with the higher number of ABCB5^pos^ cells (Fig. 1), the higher number of Rh123^low^ stem-like cells (Fig. 2A), the lower proportion of Rh123^low^ cells that arrested in the G0 phase of the cell cycle, and the higher proportion of cells in the G1 phase in the DiI^high^ OCR_OCMM2 melanoma cells (Fig. 2B). As shown in Fig. 4B and C, a slight difference was observed between the kinetic of the tumor growth depending of the cell line. Tumor growth generated from sorted DiI^high^ OCR_OCMM2 cells was slower than DiI^high^ OCR_OCMM1 conditions. These observations were in agreement with our previous study [11] where the OCR_OCMM2 cells generated tumors in immunodeficient mice, later and slower compared with OCR_OCMM1 cells. When mice developed tumors with DiI^low^ OCR_OCMM1 cells, no difference was observed regarding the kinetic of the tumor growth compared with DiI^high^ OCR_OCMM1 cells (Fig. 4B).

We next performed immunostaining on tumor sections generated from sorted DiI^high^ cells. As shown in Fig. 4D, tumors expressed the differentiated melanoma cell markers, Melan-A and M-CAM. These results indicate that our canine melanoma cell lines contained quiescent/slow-cycling stem-like capable of initiating tumors in vivo and that the tumorigenic potential was cell strain-dependent.

## Discussion

In this study, we demonstrated that our canine melanoma cell lines contain a small subset of cells with SC-like activities, including a superior ability to self-renew and to regenerate tumor-like spheres with progeny that recapitulated the phenotypic heterogeneity of the parental tumors. These cells also overexpressed the ABCB5 transporter, which is a major human melanoma CSC marker. In addition, as we have previously shown in human melanoma [4], Rh123^low^ cells are capable of generating in vitro tumor-like spheres during several successive generations, and the DiI LRC SC-like subpopulation induced melanoma xenograft formation in vivo, whereas their respective counterpart did not do so nor did so with significantly less efficiency.

Numerous studies have shown the existence of cancer stem-like cells in several types of canine neoplasms [20–24], but few studies have been performed on melanoma [25]. Importantly, our in vitro and in vivo study confirmed the presence of melanoma SCs in canine melanomas.

As shown in our previous study in human melanoma [4], the size of the Rh123^low^ compartment in canine melanoma cell lines can expand in response to in vivo-like conditions, such as in melanospheres, and to the inhibition of the PI3K/AKT signaling pathway in vitro. This Rh123^low^ cell expansion that resulted from a phenotypic switch has already been described in melanomas [2,26]. Our experiments with LY294002, an inhibitor of the PI3K/AKT-signaling pathway, strongly implied that a phenotypic switch, rather than a symmetric CSC division mechanism, was involved in Rh123^low^ cell expansion. We found that in response to short-term LY294002 treatment, a number of plastic Rh123^high^ cells switched to the Rh123^low^ phenotype, expanding the Rh123^low^ cell compartment.

As previously shown in human melanoma [4], PI3K/AKT signaling maintains G0-Rh123^low^ SCs in a quiescent state but promotes the exit of active Rh123^low^ cells in the G1 state from the SC compartment by modulating FOXO3a functionality. Additionally, in our B16 mouse model of melanoma dormancy [5], we showed that persistent tumor cells that escaped cancer vaccine therapy displayed SC-like properties and that the balance between their quiescence and proliferation was controlled by FOXO3a, a downstream target of AKT. In addition, our recent study [11] showed that both OCR_OCMM1 and OCR_OCMM2 canine melanoma cells displayed high levels of PI3K/AKT pathway activity, and genomic alterations (loss of CNAs) in *PTEN* tumor suppressor genes were observed in OCR_OCMM2 cells. The PI3K/AKT pathway can be activated by mutations in the *PTEN* gene and by the loss of PTEN protein expression, and these events have already been observed in canine and human melanomas [27]. Other studies have shown similarly high levels of PI3K/AKT pathway activity in primary canine melanoma [28,29]. These findings in canine, murine, and human melanoma models reinforce the crucial role of the PI3K/AKT signaling pathway not only in melanoma development but also in controlling the size of the CSC compartment. The accumulation of similar data in canine mucosal and human cutaneous melanoma cell lines suggests the generality (universality) of these findings, regardless of the tissue origins of melanoma, that is, cutaneous or mucosal.

In this study, we observed a significant difference between OCR_OCMM1 and OCR_OCMM2 canine melanoma cell lines regarding the size and behavior of the CSC compartment, as identified by the Rh123^low^ or ABCB5^pos^DiI^high^ phenotypes. Indeed, in the OCR_OCMM2 cell line, the SC compartment was significantly larger, was highly enriched with stem-like cells, and appeared to be less susceptible to phenotypic switching than in the OCR_OCMM1 cell line. These results could be correlated with the clinical melanoma profiles in the two dogs from which primary tumors have been extracted [11]. Indeed, the OCR_OCMM2 cell line was derived from a dog with melanoma and lung metastasis, whereas the OCR_OCMM1 cell line was derived from a dog with melanoma with no metastasis.

These results agree with the previous data, including ours, which have already shown that there is a correlation among aggressiveness, metastatic development, and the size of the CSC compartment [30,31]. Interestingly, our data suggest that metastatic development may be related to the proportion of G0 quiescent versus active G1 cells in the SC compartment. These differences in the clinical and biological manifestations between the two cell lines may also be related to differences in the genomic alterations identified by comparative genomic hybridization arrays [11]. Whereas no crucial genes associated with SC identity were altered in these cells, genes from major pathways implicated in (i) the regulation of CSCs, such as PTEN through PI3K/AKT [4], or (ii) the regulation of the cell cycle, such as CDKN2A or p16^INK4a^ [32,33], were altered at the genetic level [11].

These results could also explain the slight distinction in the behavior of the CSC compartments in response to the inhibition of the PI3K/AKT pathway. Indeed, the OCR_OCMM1 stem-like compartment was significantly larger than the OCR_OCMM2 SC pool following LY294002 treatment. Since OCR_OCMM2 cells, but not OCR_OCMM1 cells, did not have functional PTEN and p16^INK4a^, the observed differences in the phenotypic switch may be PTEN- and/or p16^INK4a^-dependent. Our two in vitro models of melanoma CSCs could therefore be useful for studying CSC biology because of their different phenotypes that involve key SC regulators, as described previously. Importantly, since these two canine melanoma cell lines displayed distinct genomic alterations, they may help to decipher the complex regulation of the CSC compartment.

In conclusion, we determined that our canine melanoma cell lines contain a subpopulation of Rh123^low^ stem-like cells in a quiescent state and ABCB5^pos^DiI^high^ LRCs, similar to human melanoma, and that the PI3K/AKT signaling pathway regulates the size of this cell compartment. Collectively, these results helped to enhance the understanding of melanoma tumor biology, making the newly isolated OCR_OCMM1 and OCR_OCMM2 canine melanoma cell lines powerful tools to study the quiescent CSC compartment. These two cell lines displayed distinct behaviors in regard to the CSC compartment size and/or expansion and could be used to test new targeted therapeutic strategies.

## Supplementary Material

Supplemental data

Supplemental data
