# Supplementary material for: Aggressiveness Potential of Spontaneous Canine Mucosal Melanoma Can Dictate Distinct Cancer Stem Cell Compartment Behaviors in Regard to Their Initial Size and Expansion Abilities
Source: Stem Cells Dev. 2020 Jul 9;29(14):919–28. doi: 10.1089/scd.2019.0223 (PMC7374591; doi:10.1089/scd.2019.0223)
Supplement: Supplemental data [file Supp_FigS1.pdf]

Supplementary Data

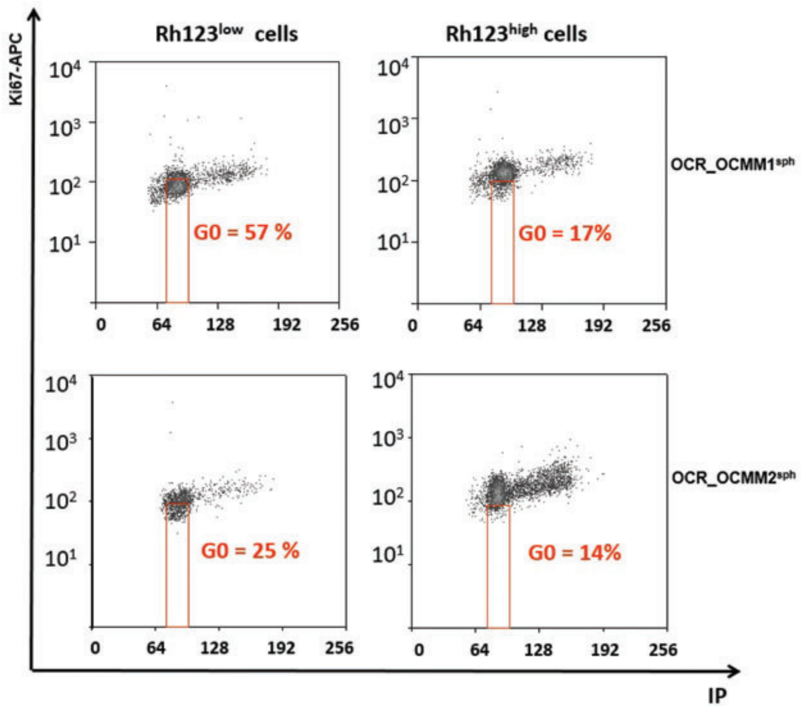

SUPPLEMENTARY FIG. S1. Rh123^{low} cells from spheroids are enriched in quiescent G0 cells in both canine melanoma cell lines. Flow cytometry Ki67/PI assay with FACS-sorted Rh123^{low} and Rh123^{high} OCR_OCMM1 and OCR_OCMM2 spheroid cells (*red box* represents the percentage of G0 cells), showing the percentage of G0 cells. FACS, fluorescence-activated cell sorting; PI, propidium iodide; Rh123, rhodamine 123.
