# Supplementary material for: Aggressiveness Potential of Spontaneous Canine Mucosal Melanoma Can Dictate Distinct Cancer Stem Cell Compartment Behaviors in Regard to Their Initial Size and Expansion Abilities
Source: Stem Cells Dev. 2020 Jul 9;29(14):919–28. doi: 10.1089/scd.2019.0223 (PMC7374591; doi:10.1089/scd.2019.0223)
Supplement: Supplemental data [file Supp_FigS2-S3.pdf]

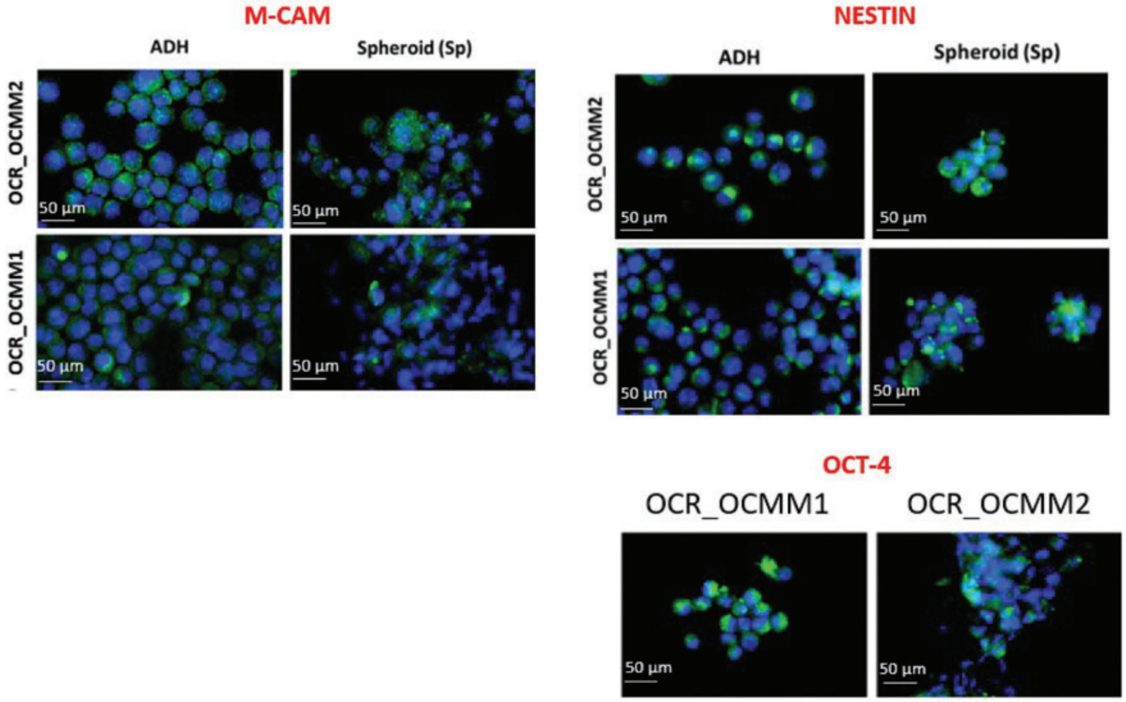

SUPPLEMENTARY FIG. S2. Melanospheres generated in vitro resemble the parental cell line-derived tumor. A similar pattern of M-CAM, Nestin, and OCT4 expression was observed as determined by immunohistochemistry using the corresponding Abs described in the Materials and Methods section. Nuclei were stained with DAPI. Scale bar, 50 µm. DAPI, diamidino-2-phenylindole; Abs, antibodies.

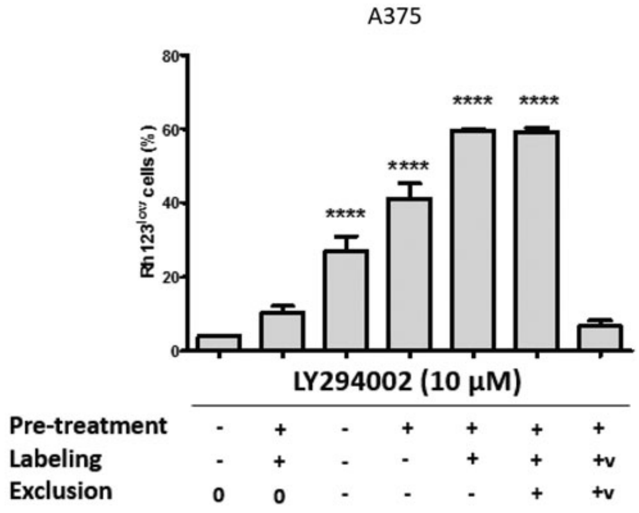

SUPPLEMENTARY FIG. S3. Time- and ABC transporter-dependent effects of LY294002 on the size of the Rh123^{low} pool in the human A375 melanoma cell line. The cells were treated with 10 µM of LY294002 (+) or growth medium (–) for different durations during the Rh123 dye exclusion assay (pretreatment-30 min; labeling-20 min; and exclusion-60 min) ($n = 8$). A final concentration of 50 µM verapamil (V) was added as a control where indicated; (0) indicates no exclusion. ****$P \leq 0.001$.
